# Comparative assessment of LDL-C and VLDL-C estimation in familial combined hyperlipidemia using Sampson’s, Martin’s and Friedewald’s equations

**DOI:** 10.1186/s12944-021-01471-3

**Published:** 2021-05-05

**Authors:** Arsenio Vargas-Vázquez, Omar Yaxmehen Bello-Chavolla, Neftali Eduardo Antonio-Villa, Roopa Mehta, Ivette Cruz-Bautista, Carlos A. Aguilar-Salinas

**Affiliations:** 1 Unidad de Investigación de Enfermedades Metabólicas, Instituto Nacional de Ciencias Médicas y Nutrición Salvador Zubirán, Mexico City, Mexico; 2 MD/PhD (PECEM) program, Faculty of Medicine, National Autonomous University of Mexico, Mexico City, Mexico; 3 Dirección de Investigación, Instituto Nacional de Geriatría, Mexico City, Mexico; 4 Departamento de Endocrinología y Metabolismo, Instituto Nacional de Ciencias Médicas y Nutrición Salvador Zubirán, Mexico City, Mexico; 5 Instituto Tecnologico y de Estudios Superiores de Monterrey Tec Salud, Mexico City, Mexico

**Keywords:** Low-density lipoprotein cholesterol, Familial combined hyperlipidemia, Very low-density lipoprotein cholesterol, Cardiovascular risk, Martin/Hopkins’ equation, Friedewald’s equation, Sampson’ equation

## Abstract

**Background:**

Sampson et al. developed a novel method to estimate very low-density lipoprotein cholesterol (VLDL-C) and low-density lipoprotein cholesterol (LDL-C) in the setting of hypertriglyceridemia. Familial Combined Hyperlipidemia (FCHL) is a common primary dyslipidemia in which lipoprotein composition interferes with LDL-C estimation. This study aimed to evaluate performance of LDL-C using this new method (LDL-S) compared with LDL-C estimated by Friedewald’s and Martin eq. (LDL-F, LDL-M) in FCHL.

**Methods:**

Data were collected from 340 subjects with confirmed FCHL. Concordance for VLDL-C measured by ultracentrifugation and LDL-C estimated using these measures compared to Sampson’s, Martin’s and Friedewald’s equations was performed using correlation coefficients, root mean squared error (RMSE) and bias. Also, concordance of misclassified metrics according to LDL-C (< 70 and < 100 mg/dL) and Apo B (< 80 and < 65 mg/dL) thresholds were assessed.

**Results:**

Sampson’s equation was more accurate (RMSE 11.21 mg/dL; R^2^ = 0.88) compared to Martin’s (RMSE 13.15 mg/dL; R^2^ = 0.875) and the Friedewald’s equation (RMSE 13.7 mg/dL; R^2^ = 0.869). When assessing performance according to LDL-C, Sampson’s had highest correlation and lowest RMSE compared to other equations (RMSE 19.99 mg/dL; R^2^ = 0.840). Comparing performance strength across triglyceride levels, Sampson’s showed consistently improved correlations compared to Martin’s and Friedewald’s formulas for increasing triglycerides and for the FCHL phenotype of mixed dyslipidemia. Sampson’s also had improved concordance with treatment goals.

**Conclusions:**

In FCHL, VLDL-C and LDL-C estimation using Sampson’s formula showed higher concordance with lipid targets assessed using VLDL-C obtained by ultracentrifugation compared with Friedewald’s and Martin’s equations. Implementation of Sampson’s formula could improve treatment monitoring in FCHL.

## Introduction

Low-density lipoprotein cholesterol (LDL-C) is the principal lipid target to reduce cardiovascular risk in the management of dyslipidemias [1, 2]. However, LDL-C is routinely calculated using standard lipid profile and its estimation is affected by lipoprotein triglyceride content [3, 4]. Familial Combined Hyperlipidemia (FCHL) is the most common primary atherogenic dyslipidemia and is characterized by very-low-density lipoprotein (VLDL) overproduction and fluctuations in the serum lipid profile, making it difficult to estimate LDL-C in clinical settings [5]. FCHL is characterized by three phenotypes; isolated hypercholesterolemia, mixed dyslipidemia and isolated hypertriglyceridemia, and particularly is accompanied by elevated apolipoprotein B (Apo B) levels. Also, a fluctuating lipid profile and variable lipoprotein expression are commonly seen during clinical follow-up, even in the same patient [6]. Several studies have shown that in FCHL, the qualitative properties of lipoproteins are altered, including chemical composition and characteristics of VLDL, LDL and High-density lipoprotein (HDL). These alterations are more evident in subjects with hypertriglyceridemia phenotype. Therefore, the estimation of LDL-C or cholesterol in other lipoproteins using non-direct methods or equations could be biased [7, 8].

Whereas Friedewald’s equation considers a fixed ratio of triglycerides (TG): to VLDL-C of 5:1, Martin’s equation considers the interindividual variance of this ratio across different triglycerides and non-HDL cholesterol (non-HDL-C) concentrations that resulted in an adjustable factor to determinate a strata-specific median TG: VLDL-C ratio, (non-HDL-C – TG/adjustable factor). This approach resulted in a greater concordance with the measurement of LDL-C by ultracentrifugation [9, 10] Recently, Sampson et al. developed a novel method to improve LDL-C estimation in the setting of hypertriglyceridemia and/or low LDL-C. Using β-quantification results from a population with high frequency of hypertriglyceridemia, they first improved VLDL-C estimation and then used the VLDL-C equation to improve LDL-C estimation using non-linear modeling. These improvements in estimation reduce the likelihood of obtaining negative values in the setting of very low LDL-C values or in patients with mixed dyslipidemia or isolated hypertriglyceridemia [11].. However, this novel method has not been validated in patients with large fluctuations in the lipid profile as it occurs in FCHL, offering a unique opportunity to assess its performance in a population with high cardiovascular risk. Using VLDL-C measured by ultracentrifugation and LDL-C estimated using these VLDL-C measures, the correlation and concordance of VLDL-C and LDL-C, as calculated with Martin’s, Friedewald’s and Sampson’s equations, with VLDL-C and LDL-C were assessed in patients with FCHL. Furthermore, the correct classification of patients in different LDL-C treatment groups were evaluated to assess its impact in assessing reductions in cardiovascular risk.

## Material and methods

### Study population

Subjects attending the lipid Clinic at the Instituto Nacional de Ciencias Médicas y Nutrición, Salvador Zubirán in Mexico Citywith previous diagnosis of familial combined hyperlipidemia (FCHL) were included. FCHL was diagnosed using the following criteria: Apo B level > 90th percentile for Mexican population (> 108 mg/dl for men and > 99 mg/dl for women) and hypercholesterolemia (total cholesterol > 200 mg/dl) and/or hypertriglyceridemia (triglycerides > 150 mg/dl) along with the demonstration of dyslipidemia in three first-degree relatives [5]**.** Exclusion criteria included VLDL-C (mmol/L)/triglycerides (mmo/L) ratio > 0.69 and Apo B < 90th percentile (type III hyperlipoproteinemia) [12], history of an acute illness within previous six weeks, pregnancy and the presence of any disease or medication known to significantly influence lipid parameters. The Human Research Ethics Committee of the Instituto Nacional de Ciencias Médicas y Nutrición Salvador Zubirán approved all proceedings related to the study and all participants gave written informed consent. All methods and procedures were done in accordance with the Declaration of Helsinki.

### Laboratory measurements

Blood samples were obtained after 8–12 h fast. Cholesterol, triglycerides, HDL cholesterol and apolipoprotein B were measured in serum using colorimetric assays (Unicel DxC 600 Synchron Clinical System Beckman Coulter). VLDL lipoproteins were isolated using sequential ultracentrifugation (Optimal Beckman LE80-K) of 40,000 RPM at 4 °C for 18 h. Serum aliquots (3.5 mL) were centrifuged at background density of 1.006 Kg/L, VLDL-C and VLDL-triglycerides levels in the ultracentrifugal bottom fraction were analyzed by calorimetric assays (Unicel DxC 600 Synchron Clinical System Beckman Coulter). LDL and VLDL cholesterol were calculated using the Friedewald’s equation (VLDL-F, LDL-F), Sampson’s method (VLDL-S, LDL-S) and the calculation proposed by Martin et al. (VLDL-M, LDL-M). LDL-C was also calculated using VLDL-C measures by ultracentrifugation to approximate a gold-standard for comparative assessments.

### Statistical analyses

Data are presented as frequencies and percentage for qualitative variables and mean ± standard deviation or as median and interquartile range for quantitative variables. To compare proportions and medians between groups, chi-square test and Mann-Whitney-U tests were performed. Spearman correlations were performed to evaluate the degree of linear association between VLDL-C, VLDL-S, VLDL-Martin and VLDL-F. To estimate degree of linear fit R2 and the squared root of the mean squared error (RMSE) were used to estimate deviances from VLDL-C or LDL-C measured or estimated by ultracentrifugation, respectively. Concordance between LDL-C estimated using VLDL-C by ultracentrifugation as:

$$ LDL-C= TC-\left[ HDL-C+ VLDL-C\right]\Big) $$

LDL-M, LDL-S and LDL-F targets were dichotomized for each patient and compared to targets obtained by LDL-C estimated using VLDL-C by ultracentrifugation using the kappa coefficients and bias (*d*) was estimated using Bland-Altman analyses only in individuals with triglycerides < 800 mg/dL; a sensitivity analysis was performed only in subjects who had previous statin treatment to estimate significant deviances. Also, correlations and concordance of lipid goals according to the differing phenotypes of FCHL were evaluated, namely isolated hypercholesterolemia and mixed dyslipidemia. Performance of each estimation method for lipid goals of LDL-C < 100 mg/dL, LDL-C < 70 mg/dL, Apo B < 80 mg/dL and Apo B < 65 mg/dL were evaluated using areas under the receiving operating characteristic curve (AUC). A two-tailed *p*-value < 0.05 was considered significant as statistically significant. Statistical analyses were performed using the SPSS software (version 24.0) and R software (Version 3.6.2, https://www.R-project.org) [13].

## Results

### Study population

In total, 340 subjects with confirmed FCHL diagnosis and available VLDL-C measures were included. The median age of patients at diagnosis was 47.0 (35.0–58.0) years, 65% were women, 12.0% were under statin treatment and 19.11% had type 2 diabetes (T2D). Overall, 137 (40.3%) subjects who satisfied the diagnosis of isolated hypercholesterolemia and 203 (59.7%) who belonged to the mixed dyslipidemia phenotype were identified, it was not observed subjects who completed criteria for isolated hypertriglyceridemia. On comparing differences across FCHL phenotypes, in the mixed dyslipidemia phenotype the age at diagnosis was highest, fewer patients were women, more often had T2D and more patients were under statin treatment compared to the isolated hypercholesterolemia phenotype (*P* <  0.010). As expected, subjects with mixed dyslipidemia had higher values of apolipoprotein B, non-HDL cholesterol, LDL-C and VLDL-C (*P* <  0.001, Table 1).

**Table 1:** Biochemical and clinical characteristics of patients with FCHL in the overall population and stratified by FCHL dyslipidemia phenotype

**Variable**	**Overall** ***n* = 340**	Isolated hypercholesterolemia ***n* = 137**	**Mixed dyslipidemia** ***n* = 203**	***P***
Sex (female)	221 (65.0)	105 (76.6)	116 (57.1)	< 0.001
Age (years)	47.0 (35.0–58.0)	43.0 (32.0–57.0)	48.0 (37.0–58.0)	0.019
Type 2 Diabetes (%)	65 (19.1)	10 (7.3)	55 (27.1)	< 0.001
Hypertension (%)	70 (20.6)	21 (15.3)	49 (24.3)	0.046
Total cholesterol (mg/dL)	209.0 (179.0–241.5)	179.0 (160.0–198.8)	226.5 (206.0–266.8)	< 0.001
HDL cholesterol (mg/dL)	42.0 (35.0–48.8)	47.0 (41.0–54.0)	38.0 (33.0–44.0)	< 0.001
Non-HDL cholesterol (mg/dL)	168.0 (133.0–198.0)	129.5 (112.0–154.8)	188.0 (168.3–227.0)	< 0.001
Triglycerides (mg/dL)	182.5 (107.3–310.3)	99.0 (73.0–122.3)	271.0 (205.5–394.8)	< 0.001
Apolipoprotein B (mg/dL)	116.0 (90.0–136.8)	87.0 (72.9–103.8)	128.5 (114.3–148.8)	< 0.001
VLDL-Triglycerides (mg/dL)	120.8 (61.3–240.1)	54.7 (34.0–73.9)	211.0 (144.2–329.9)	< 0.001
VLDL-Cholesterol (mg/dL)	32.4 (16.5–52.5)	14.7 (9.1–19.7)	49.0 (36.1–67.4)	< 0.001
VLDL-Cholesterol Martin (mg/dL)	32.9 (21.3–46.0)	19.7 (16.0–22.9)	43.5 (34.5–56.4)	< 0.001
VLDL-Cholesterol Sampson (mg/dL)	35.1 (18.6–54.4)	16.5 (12.0–20.9)	51.6 (37.8–69.7)	< 0.001
VLDL-Cholesterol Friedewald (mg/dL)	36.5 (21.5–62.1)	19.8 (14.6–24.5)	54.2 (41.1–79.0)	< 0.001
LDL-Cholesterol (mg/dL) *	127.7 (106.4–151.6)	114.2 (98.4–136.9)	139.0 (119.4–161.7)	< 0.001
LDL-cholesterol Martin (mg/dL)	130.1 (106.2–151.4)	109.4 (95.0–131.4)	142.9 (121.5–162.3)	< 0.001
LDL-Cholesterol Sampson (mg/dL)	127.8 (101.1–145.8)	111.8 (95.6–133.1)	134.6 (110.7–157.5)	< 0.001
LDL-Cholesterol Friedewald (mg/dL)	122.6 (97.2–142.4)	109.4 (94.4–130.9)	128.8 (102.1–153.5)	< 0.001
Statin treatment (%)	41 (12.0)	5 (3.6)	36 (17.7)	< 0.001

### VLDL-C comparative assessment

**Fig. 1:**
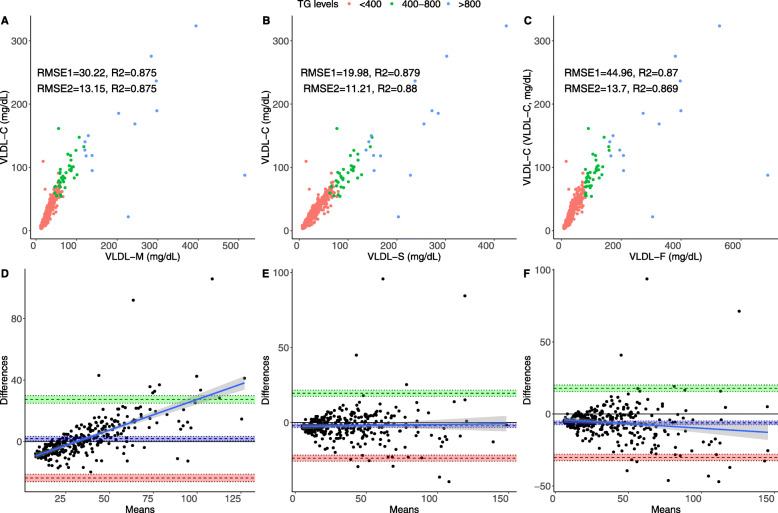
Performance metrics for all three formulas compared to VLDL-C measured by ultracentrifugation in the overall population, showing RMSE for the overall population (RMSE1, *n* = 340) and for subjects with triglycerides < 800 mg/dL (RMSE2) comparing VLDL-C measured by Martin’s (**a**), Sampson’s (**b**) and the Friedewald’s equation. The figure also shows Bland-Altman plots showing bias and limits of agreement for VLDL-C estimated using Martin’s (**d**), Sampson’s (**e**) and the Friedewald’s equation (**f**). Abbreviations = RMSE: Root of Mean Squared Error; 95%CI: 95% Confidence Interval; LDL-F: LDL-C estimated by the Friedewald’s equation; LDL-M: LDL-C estimated by Martin’s formula; LDL-S: LDL-C estimated by Sampson’s formula

For VLDL-C measured by ultracentrifugation, Sampson’s formula had the highest correlation for estimated VLDL-C (ρ = 0.937, 95%CI 0.921–0.948), followed by Martin’s (ρ = 0.935, 95%CI 0.921–0.948) and Friedewald’s (ρ = 0.933, 95%CI 0.917–0.945) formulas. VLDL-C estimation errors (RMSE) were also comparatively lower for Sampson’s formula, followed by Martin’s and Friedewald’s and were further reduced when only analyzing individuals with triglycerides < 800 mg/dL (Fig. 1**A-C**). Bland-Altman analyses showed smaller bias for Martin’s formula (*d =* 1.87, 95%CI 0.46,3.30) followed by Sampson’s (*d =* − 2.09, 95%CI -3.29-0.90) and Friedewald’s formulas (*d = − 6.20,* 95%CI 0.45–3.30, Figs. 1**D-F**) compared to VLDL-C measured by ultracentrifugation.

### LDL-C comparative assessment

**Fig. 2:**
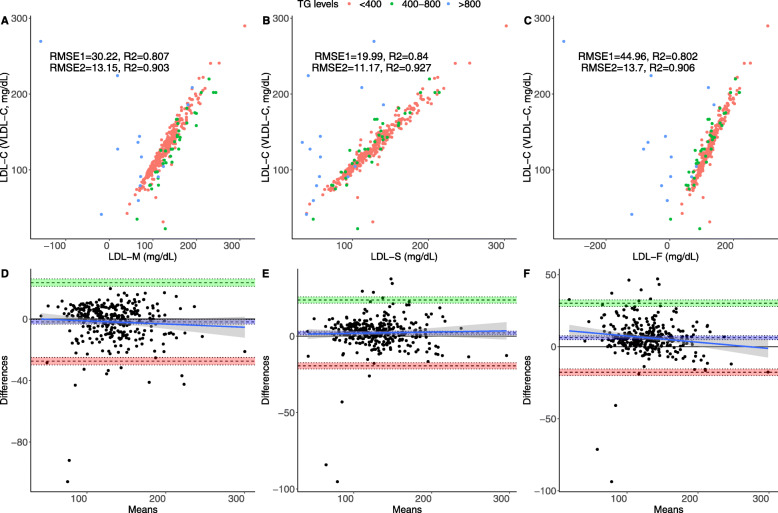
Performance metrics for all three formulas compared to LDL-C estimated using VLDL-C measured by ultracentrifugation in the overall population, showing RMSE for the overall population (RMSE1, *n* = 340) and for subjects with triglycerides < 800 mg/dL (RMSE2) comparing VLDL-C measured by Martin’s (**a**), Sampson’s (**b**) and the Friedewald’s equation. The figure also shows Bland-Altman plots showing bias and limits of agreement for VLDL-C estimated using Martin’s (**d**), Sampson’s (**e**) and the Friedewald’s equation (**f**). Abbreviations = RMSE: Root of Mean Squared Error; 95%CI: 95% Confidence Interval; LDL-F: LDL-C estimated by the Friedewald’s equation; LDL-M: LDL-C estimated by Martin’s formula; LDL-S: LDL-C estimated by Sampson’s formula

For LDL-C, Sampson’s formula had the highest correlation, which also displayed the lowest RMSE and highest R^2^ despite having slightly higher bias compared to Martin’s (Table 2**,** Fig. 2); similarly, Sampson’s formula had lower bias compared to Martin’s and Friedewald’s formulas for LDL-C estimation. When assessing performance according to dyslipidemia phenotypes, Friedewald’s and Sampson’s formulas had similar RMSE and linear correlation, which were higher than Martin’s for isolated hypercholesterolemia. However, performance of Sampson’s formula drastically improved in mixed dyslipidemia compared to other methods. Comparing correlation strength across triglyceride levels, Sampson’s showed consistently improved correlations compared to Martin’s and Friedewald’s formulas for triglyceride categories < 400 mg/dL [LDL-S: ρ = 0.962, 95% CI 0.952–0.970; LDL-M: ρ = 0.956, 95%CI 0.945–0.965; LDL-F: ρ = 0.955, 95%CI 0.944–0.964] and even >400mgdL [LDL-S: ρ = 0.642, 95%CI 0.445–0.779; LDL-M: ρ = 0.508, 95%CI 0.270–0.687; LDL-F: ρ = 0.577, 95%CI0.359–0.736]. Nevertheless, Sampson’s formula had slightly higher bias compared to Martin’s when compared using Bland-Altman analyses. *Apo B comparative assessment.*

**Table 2:** Performance metrics for all three formulas compared to LDL-C estimated using VLDL-C measured by ultracentrifugation in the overall population and stratified by FCHL dyslipidemia phenotype

**Metric**	**LDL-F**	**LDL-M**	**LDL-S**	**Isolated Hypercholesterolemia**			**Mixed dyslipidemia**		
				LDL-F	LDL-M	LDL-S	LDL-F	LDL-M	LDL-S
ρ (95%CI)	0.895 (0.872–0.915)	0.899 (0.876–0.917)	0.917 (0.899–0.932)	0.962 (0.947–0.973)	0.957 (0.941–0.969)	0.961 (0.9460.972)	0.855 (0.814–0.889)	0.871 (0.834–0.901)	0.875 (0.838–0.904)
ρ with ApoB (95%CI)	0.644 (0.577–0.702)	0.788 (0.744–0.825)	0.704 (0.646–0.754)	0.856 (0.825–0.882)	0.868 (0.8400.892)	0.862 (0.832–0.887)	0.628 (0.558–0.688)	0.729 (0.675–0.775)	0.662 (0.598–0.718)
R2	0.802	0.807	0.840	0.645	0.606	0.614	0.731	0.769	0.782
RMSE	44.96	30.22	19.99	10.74	10.98	10.19	44.44	29.41	18.41
Bias (95%CI)	12.33 (7.71,16.95)	1.12 (−2.10,4.35)	4.59 (2.51,6.67)	2.85 (2.16,5.56)	4.14 (2.41,5.86)	2.29 (0.59,4.00)	18.05 (10.47,25.63)	−0.91 (−6.19,4.37)	6.14 (2.86,9.42)

*Abbreviations*: *RMSE* Root of Mean Squared Error, *95%CI* 95% Confidence Interval, *LDL-F* LDL-C estimated by the Friedewald’s equation, *LDL-M* LDL-C estimated by Martin’s formula., *LDL-S* LDL-C estimated by Sampson’s formula

To compare the correlations between Apo B and LDL-C estimated by the three equations, Martin’s formula had the highest correlation for overall and in isolated hypercholesterolemia, but Sampson’s had slightly higher correlation in mixed dyslipidemia compared to Martin’s equation (Table 2). However, comparing correlations strength across triglyceride levels, Martin’s showed consistently improved correlations compared to Sampson’s and Friedewald’s formulas for triglyceride categories < 400 mg/dL [LDL-M: ρ = 0.853, 95%CI0.818–0.881; LDL-S: ρ = 0.806, 95%CI 0.761–0.843; LDL-F: ρ = 0.772, 95%CI 0.721–0.815] and 400-800 mg/dL (LDL-M: ρ = 0.853, 95%CI 0.727–0.924; LDL-S: ρ = 0.843, 95%CI 0.710–0.918; LDL-F: ρ = 0.836, 95%CI 0.697–0.914). Overall, Sampson’s formula had the highest correlation compared to the other equations for triglycerides > 800 mg/dL (LDL-S: ρ = 0.586, 95%CI 0.081–0.852; LDL-M: ρ = 0.088, 95%CI -0.464-0.590; LDL-F: ρ = 0.082, 95%CI -0.468-0.587).

### Comparison of LDL-C formulas for LDL-C and Apo B targets

When assessing concordance in lipid target goals (LDL-C < 100 mg/dL), the highest concordance and AUROC were observed for Sampson’s formula overall and in both isolated hypercholesterolemia and mixed dyslipidemia (Table 3). For more stringent lipid targets (LDL-C < 70 mg/dL) Sampson’s formula had lower concordance compared to Martin’s, but higher AUROC, which was comparable to Friedewald’s equation in isolated hypercholesterolemia. In mixed dyslipidemia, Martin’s equation had highest concordance, but Sampson’s formula had highest AUROC. This is consistent with previous findings relating LDL-M in FCHL. Finally, when assessing concordance in lipid target goals (Apo B < 80 mg/dL) the highest concordance and AUROC were observed for Martin’s formula overall and in both isolated hypercholesterolemia and mixed dyslipidemia. For more stringent lipid targets (Apo B < 65 mg/dL), Martin’s showed consistently improved concordances and AUROC compared to Sampson’s and Friedewald’s formulas overall and in both phenotypes.

**Table 3:** Comparison of lipid targets for all three formulas compared to LDL-C estimated using VLDL-C measured by ultracentrifugation in the overall population and stratified by FCHL dyslipidemia phenotype

**Metric**		**LDL-F**	**LDL-M**	**LDL-S**	**Isolated Hypercholesterolemia**			**Mixed dyslipidemia**		
					**LDL-F**	**LDL-M**	**LDL-S**	**LDL-F**	**LDL-M**	**LDL-S**
LDL-C goal < 100 mg/dL	κ (95%CI)	0.730 (0.642–0.818)	0.764 (0.674–0.854)	0.819 (0.740–0.997)	0.723 (0.630–0.843)	0.752 (0.636–0.868)	0.779 (0.666–0.892)	0.709 (0.568–0.850)	0.731 (0.566–0.897)	0.852 (0.735–0.968)
	AUC (95%CI)	0.923 (0.884–0.961)	0.892 (0.835–0.948)	0.933 (0.890–975)	0.898 (0.839–0.956)	0.909 (0.851–0.965)	0.911 (0.851–0.970)	0.938 (0.883–0.994)	0.815 (0.690–0.940)	0.943 (0.871–1.00)
LDL-C goal < 70 mg/dL	κ (95%CI)	0.338 (0.127–0.549)	0.506 (0.252–0.759)	0.462 (0.216–0.707)	0.560 (0.115–1.00)	0.453 (0.012–0.839)	0.560 (0.115–1.00)	0.279 (0.054–0.504)	0.557 (0.242–0.872)	0.424 (0.136–0.712)
	AUC (95%CI)	0.870 (0.750–0.990)	0.869 (0.742–0.995)	0.878 (0.759–0.997)	0.730 (0.412–1.00)	0.727 (0.405–1.00)	0.731 (0.409–1.00)	0.942 (0.877–1.00)	0.929 (0.816–1.00)	0.951 (0.877–1.00)
ApoB goal < 65 mg/dL	κ (95%CI)	0.081 (−0.066–0.228)	0.269 (0.059–0.478)	0.127 (− 0.047–0.301)	0.159 (− 0.054–0.371)	0.308 (0.063.0.553)	0.159 (− 0.054–0.371)	0.07 (− 0.082–0.230)	0.187 (−0.143–0.517)	0.128 (− 0.115,0.371)
	AUROC (95%CI)	0.869 (0.815–0.922)	0.915 (0.858–0.971)	0.898 (0.841–0.954)	0.931 (0.882–0.980)	0.935 (0.887–0.982)	0.935 (0.888–0.982)	0763 (0.394–1.00)	0.775 (0.373–1.00)	0.763 (0.325–1.00)
ApoB goal < 80 mg/dL	κ (95%CI)	0.450 (0.342–0.558)	0.570 (0.459–0.681)	0.463 (0.347–0.579)	0.674 (0.547–0.802)	0.672 (0.554–0.800)	0.605 (0.467–0.742)	0.024 (−0.044,0.091)	0.060 (−0.071,0.190)	0.039 (− 0.056,0.137)
	AUROC (95%CI)	0.827 (0.785–0.872)	0.905 (0.870–0.939)	0.867 (0.827–0.906)	0.910 (0.863–0.957)	0.918 (0.873–0.963)	0.913 (0.867–0.959)	0.762 (0.394–1.00)	0.775 (0.373–1.00)	0.763 (0.325–1.00)

*Abbreviations*: *AUROC* Area Under the ROC Curve, *95%CI* 95% Confidence Interval, *LDL-F* LDL-C estimated by the Friedewald’s equation, *LDL-M* LDL-C estimated by Martin’s formula, *LDL-S* LDL-C estimated by Sampson’s formula

## Discussion

Liver production of lipoproteins and its lipid content, particularly in the case of VLD-C, is markedly altered in patients with FCHL [5]. Recently, our group performed an external validation of Martin’s formula in FCHL demonstrating an improved performance for this method compared to apolipoprotein B and non-HDL cholesterol in concordance and misclassification of treatment goals [14]. Despite the utility of Martin’s formula, the pathophysiology of FCHL with a concurrent insulin resistant state, increased lipolysis and variable expression of triglyceride-variants in this condition offers variable increases in triglyceride concentration, diminishing the utility of this formula as LDL-C is modified by treatment in the setting of hypertriglyceridemia and mixed dyslipidaemia. Furthermore, FCHL patients tend to have more dysfunctional atherogenic lipoproteins and thus a higher incidence of cardiovascular disease, which might require higher intensity treatment and would benefit from improved LDL-C estimation [7, 15].

The novel method proposed by Sampson et al. offers an attractive alternative to estimate VLDL-C and LDL-C in the setting of lipid profile fluctuations, particularly in cases of hypertriglyceridemia and lowering LDL-C for treatment reassessment. This allows for more precise assessment of cardiovascular risk management in FCHL by improving prediction of VLDL-C, the most variable component in LDL-C estimation, whilst also potentially allowing for more accurate estimation of remnant cholesterol [16]. As an illustrative example of the utility of the different LDL-C formulas, consider the case of a patient with lipid profile within our study, who had triglycerides at 1986 mg/dL, total cholesterol 299 mg/dL and HDL-C 21 mg/dL. LDL-C calculated using VLDL-C measurement was 41.46 mg/dL, whereas LDL-C estimated with the 3 formulas were LDL-F − 119.2 mg/dL, LDL-M − 18.418 mg/dL and LDL-S 39.311 mg/dL. In this context, LDL-C estimation with Friedewald’s and Martin’s formulas result in a negative value that it is not plausible, whilst Sampson’s equation performed a value closer to the LDL-C estimated with VLDL-C measurement by ultracentrifugation method.

This result demonstrates that VLDL-C and LDL-C estimated using Sampson’s equation is a better estimator over the traditional Friedewald’s and Martin’s formulas, showing a significantly higher correlation and agreement with VLDL-C measured by ultracentrifugation and LDL-C estimated using these VLDL-C measures in subjects with FCHL. Even in the setting of hypertriglyceridemia, which is frequent in FCHL and might significantly fluctuate through the course of the disease, Sampson’s equation is still significantly better than other formulas. When analysing FCHL phenotypes, LDL-C estimated using Sampson’s and Friedewald’s equations perform similarly in the setting of isolated hypercholesterolemia; however, Sampson’s formula had a better performance in the setting of mixed dyslipidaemia. Population-based research in the US and Korea has shown that improved LDL-C estimation might offer more precise assessment of treatment goals and allow for better informed treatment intensification which might be particularly helpful in FCHL [17, 18]. Even though Sampson’s method might underperform with triglyceride levels > 800 mg/dL, our data shows that it still holds adequate performance and is superior to Martin’s and Friedewald’s methods, indicating a use in phenotypes of isolated hypertriglyceridemia with low LDL-C values.

Therefore, improving the LDL-C estimation in a setting of hypertriglyceridemia or mixed dyslipidaemia might improve the identification of subjects under lipid-lowering treatment who would benefit to add a second drug to achieve the LDL-C goal. Indeed, achieving lower LDL-C levels is associated with a higher rate of atherosclerotic plaque regression compared to patients with more elevated LDL-C [19]. Then, the combination of lipid-lowering drugs in patients with insufficient LDL-C reduction or with high residual risk reduces the progression of coronary atherosclerosis and the risk of coronary events [19].

Apo B is highly correlated with LDL-C and non-HDL-C levels; however, Apo B is more accurate as a marker of cardiovascular risk over cholesterol and triglyceride measures, with several studies confirming these findings [20–23]. Therefore, by evaluating the correlation between Apo B levels and LDL-C estimated by these three methods, LDL-C estimated by Sampson’s equation showed the highest correlation in mixed dyslipidaemia, even for triglycerides > 800 mg/dL compared to Martin’s formula, which had shown an adequate correlation in patients with mixed dyslipidaemia and hypertriglyceridemia in a previous study [9]**.** Also**,** the performance for assessing concordance in lipid target goals (Apo B < 80 mg/dL and < 65 mg/dL) and Martin’s equation showed consistently improved concordances and AUROC compared to the other methods. However, for a given value of Apo B < 50th percentile, levels of LDL-C and non-HDL-C may range from the 25th to 75th percentile and the values will be discordant and, therefore will predict cardiovascular risk differently [24, 25]. Also, the limited number of patients under statin treatment conferred a limited number of patients with low levels of Apo B and, in this case the concordance observed between lipid target goals (Apo B < 65 and < 80 mg/dL) and LDL-C should be evaluated with reservation.

### Strengths and limitations

The study had some strengths and limitations. First, this study used VLDL-C estimation assessed using the gold standard, VLDL-C measured by Ultracentrifugation, and evaluated the performance of these equations compared to VLDL-C and LDL-C in a population with high variability in the lipid profile. Potential limitations of this approach include the non-direct method to measure LDL-C or remnant lipoproteins; to overcome this, LDL-C was calculated using VLDL-C measures by ultracentrifugation to approximate a gold-standard for comparative assessments. Similarly, the limited number of subjects with low LDL-C which is an area specifically designed for Sampson’s formula and might improve its performance compared to other methods; this may be particularly helpful whilst following up treatment efficacy and should be evaluated for FCHL and other conditions with concomitant hypertriglyceridemia. However, LDL-C estimation using Sampson’s formula is markedly more useful than traditional methods in mixed dyslipidaemia, highlighting a potential application of this formula along with Apo B assessment for cardiovascular risk management.

## Conclusions

In conclusion, Sampson’s equation might offer more precise LDL-C assessment by improving VLDL-C estimation, which might be particularly helpful in the setting of hypertriglyceridemia and mixed dyslipidaemia improving cardiovascular risk management in individuals with these phenotypes. However, the performance of Friedewald’s equation is similar to Martin’s and Sampson’s in isolated hypercholesterolemia and either method could be applicable in this setting. Finally, Sampson’s formula should be used to assess its role in improving cardiovascular risk management in FCHL and its efficacy should be evaluated during follow-up to estimate its usefulness in treatment adjustment and cardiovascular risk reduction, particularly in the setting of low LDL-C values.

## Data Availability

The dataset used and/or analysed during the current study are available from the corresponding author on reasonable request.
